# Could Ultrasonographic Measurements of the Umbilical Cord be a Predictor of Birth Weight? A Health Facility‐Based Cross‐Sectional Study

**DOI:** 10.1002/hsr2.72397

**Published:** 2026-04-16

**Authors:** Swallah Alhaji Suraka, Abdul Nashirudeen Mumuni, Nathaniel Awentiirin Angaag, Larisa‐ Ioana Coman, Eric Kwasi Ofori

**Affiliations:** ^1^ Department of Biomedical Engineering Koforidua Technical University Koforidua Eastern Region Ghana; ^2^ Department of Medical Imaging Klintaps College of Health and Allied Sciences Tema West Greater‐Accra Region Ghana; ^3^ Department of Medical Imaging, School of Allied Health Sciences University for Development Studies Tamale Northern Region Ghana; ^4^ Department of Radiology Holy Family Catholic Polyclinic Kulmasa Savannah Region Ghana; ^5^ Oxford University Hospitals NHS Foundation Trust Oxford England UK; ^6^ Department of Medical Imaging, School of Allied Health Sciences University of Health and Allied Sciences Ho Volta Region Ghana

**Keywords:** birth weight, Ghana, ultrasound, umbilical cord measurement

## Abstract

**Background:**

Factors such as maternal demographics, anthropometrics, health status, and lifestyle are known to have direct associations with newborn birth weight. However, there is an indication that umbilical cord size measured using ultrasonography could potentially be a predictor of birth weight. This study, therefore, aimed to investigate the relationship between umbilical cord measurements and birth weight in Ghana.

**Method:**

A prospective, cross‐sectional, quantitative approach was adopted for this study. A total of 107 patients were purposively sampled with an attrition rate of 9.3%, leading to 97 (90.7%) partaking in the study, aged between 17 and 41 years. The patients were recruited between 34 and 37 weeks of gestation during their routine antenatal care visit. Data was collected using a data sheet with some key parameters recorded, being: maternal age, gravida, parity, umbilical cord diameter (UCD), umbilical cord circumference (UCC) and umbilical cord area (UCA), which were manually entered into the IBM SPSS version 26.

**Results:**

The mean age recorded was 29.59 ± 4.83 years. The mean UCD, UCC and UCA were 1.53 ± 0.19 cm, 5.57 ± 0.41 cm and 1.86 ± 0.47 cm^2^, respectively. The Cronbach's alpha coefficient (*α*) was used to assess intra‐observer reliability, and it showed strong consistency and precision. The mean birth weight was 3.13 ± 0.27 kg. There were moderate positive correlations between UCD and birthweight (*r* = 0.53, *p* < 0.01), UCC and birthweight (*r* = 0.331, *p* < 0.001) and UCA and birthweight (*r* = 0.52, *p* < 0.001). There were positive correlations between maternal demographics and UCD and maternal demographics and UCA. The study found no correlation between maternal demographics and UCC. Multiple regression analysis of the predictors of birth weights in the study population was conducted. The model as a whole was significant in predicting neonatal birth weights among the study participants at F (4, 92) = 20.731, *p* < 0.001. The final predictive equation of the model for predicting BW was given as: BW = 1.849 + 0.021 × maternal age + 0.031 × parity − 0.066 × UCC + 0.652 × UCD.

**Conclusion:**

The results suggest that umbilical cord size could be an important marker for fetal weight estimation. However, the less diverse small sample size and single‐centre design of the study limit the generalizability of the findings.

## 1 Introduction

Fetal growth is influenced by many factors, including maternal hypertension, diabetes mellitus, fetal aneuploidy, as well as placental conditions such as placenta previa and velamentous cord insertion [1, 2, 3]. Throughout the perinatal period and during delivery, high or low birth weight might result in complications for the newborn [4]. Accurate fetal weight assessment before delivery is essential to anticipate and mitigate possible risks like hypoxia‐related delivery of small or excessively large foetuses [5, 6].

Prediction of birth weight can be done by clinical palpation of the maternal abdomen or by obstetric ultrasound [7]. However, the estimation of fetal weight by clinical palpation can be influenced by various factors such as amniotic fluid volume (AFV), the presence of uterine myoma(s), and maternal obesity [8]. An increase in AFV, the presence of uterine myoma(s), and the thickening of the subcutaneous tissues of the maternal abdominal wall could increase the symphysio‐fundal height, hence creating the impression of a larger fetus [7, 8]. Alternatively, assessing factors influencing fetal weight has become considerably simpler in recent years due to advancements in ultrasonography technologies. Obstetric ultrasound imaging technique provides real‐time visualization of the fetus and abdominopelvic structures, which can aid in better accuracy of estimation of fetal weight than as observed with clinical palpation [9].

The umbilical cord has been investigated in various studies, and the conclusion is that sonographic evaluation of the umbilical cord is useful in determining overall birth and perinatal outcomes [10, 11, 12] and could help in predicting birth weight. It has been suggested that an ultrasound‐based estimate of the umbilical cord diameter could be associated with birth weight [13]. According to Tutus et al. [14], umbilical cord diameter in the second trimester could be a useful measurement for predicting foetuses that are large for gestational age among Turkish pregnant women. Also, in Nigeria, Udoh et al. [15] concluded that umbilical cord diameter measurements have the potential to serve as an important indicator of fetal growth and perinatal outcome. Even though these studies have investigated the relationship between umbilical cord measurements and pregnancy outcome, especially in determining fetal weight, there are some limitations. A lack of consensus on the most reliable region of the umbilical cord (cord insertion/free loop), coupled with variations in predictive capacities across different examiners and populations, has led to inconsistencies in birth weight prediction. The potential adoption of umbilical cord diameter as a predictive tool for birth weight calls for improvements in reliability and accuracy to address the existing limitations. This study, therefore, aimed to investigate the relationship between umbilical cord measurements, birth weight, and maternal characteristics (age, gravida and parity) across term pregnant women. The study seeks to add to the body of knowledge in this subject area by providing a nomogram for umbilical cord measurements (circumference, area and diameter) and evidence of its relationship with birth weight and other maternal characteristics among a cross‐section of Ghanaian mothers.

## 2 Materials and Methods

### 2.1 Study Site

The study was conducted at the Ho Teaching Hospital in the Ho municipality of the Volta region (located in Southeastern Ghana). The hospital was purposively selected for this research because it is one of the main referral hospitals in the country, serving patients from diverse demographics and backgrounds across Southeastern Ghana. Additionally, the hospital was selected for its advanced ultrasound equipment. The hospital has a total of 13 ultrasound machines, most of them being state‐of‐the‐art, offering high‐resolution imaging and the latest obstetric software.

### 2.2 Study Design

A prospective cross‐sectional quantitative approach was used to examine the relationship between umbilical cord measurements, maternal and fetal characteristics, and birth weight. The method was adopted by analyzing multiple peer‐reviewed articles after carefully considering their strengths and weaknesses.

### 2.3 Study Population, Inclusion, and Exclusion Criteria

The target population was pregnant women who attended the antenatal clinic of the hospital. Term pregnant women with a viable single pregnancy and a gestational age of 34–37 weeks were included in the study. Women with multiple gestations, non‐black women, and those with maternal conditions and/or pregnancy‐related complications such as pre‐eclampsia, gestational diabetes, gestational hypertension and intrauterine growth restriction were excluded from the study.

### 2.4 Sample Size Determination

The sample size was determined using the G*Power software (version 3.1.9.4). The test family and statistical test selected in the software were *t*‐tests and correlation: point biserial model [16]. The minimum sample size, when calculated based on an effect size of 0.30, a significance level of 0.05, and a statistical power of 0.80, was 82. A 30% patient attrition rate after recruitment was adjusted for. The attrition rate of 30% allowed for the inclusion of patients who delivered before the scheduled ultrasound examination or those who had dropped out of the study for various reasons. Adding the calculated sample size and the anticipated attrition rate, a minimum sample size of 107 was calculated for this study.

### 2.5 Sampling Technique and Recruitment

A purposive sampling technique was used in recruiting pregnant women with the desired characteristics required for the study. The mean gestational age at birth in Ghana is 38 ± 1.9 weeks [17], which informs the rationale of having the ultrasound examinations of this study being done within 38^+0^–38^+6^ weeks. This was to ensure that the umbilical cord measurements would be taken close to birth to provide accuracy with the relationship to birth weight and provide consistency in results among the participants. The pregnant women were recruited during their routine antenatal care between 34 and 37 weeks of gestation. The recruitment was done by using the daily antenatal clinic attendance register and the maternal health records to obtain the lists of pregnant women meeting the inclusion criteria. The aim, purpose, and requirements of the study were explained to eligible pregnant women. Subsequently, those who consented to the study were scheduled for the ultrasound examination at a point within 38^+0^–38^+6^ weeks of gestation and on their scheduled day of antenatal care attendance. Recruitment using purposive sampling was done until the sample size was met.

### 2.6 Data Collection Instrument

A Vinno X1 ultrasound machine, manufactured in February 2022, was used with the obstetrics pre‐set function to obtain UCD measurements. The stock curvilinear transducer was used as its frequency range fell within the recommended frequency range (1–6 MHz) for third‐trimester obstetrics ultrasound assessment [18]. The ultrasound machine calibration was checked by the hospital's biomedical engineer as per the manufacturer's recommendations to ensure the accuracy of measurements. Additionally, a pre‐test of the ultrasound equipment's functionality was performed by the hospital's biomedical engineer using an ultrasound phantom for quality control purposes. These were done to ensure the reliability of the data. We further utilized the three‐level guidelines in performing the needed quality assurance checks on ultrasound systems by sonographers, as proposed by the British Medical Ultrasound Society (BMUS), to run additional checks on the ultrasound equipment [19].

### 2.7 Ultrasound Examination Procedure

**Figure 1:**
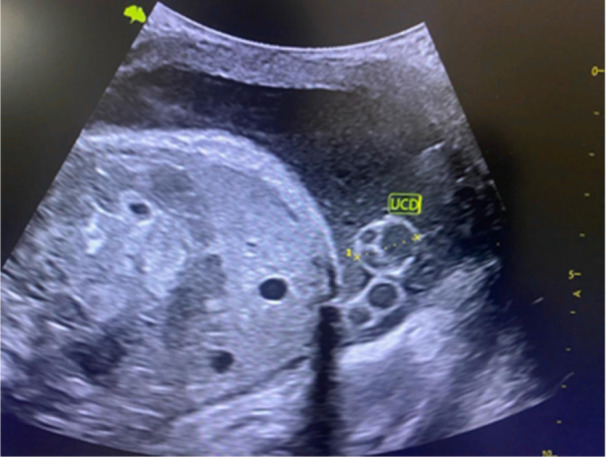
Sonographic measurement of umbilical cord diameter.

Before the examination, maternal information (age, gravida, and parity) was recorded. All ultrasound assessments were carried out within 38^+0^–38^+6^ weeks of gestation. Each participant was scanned twice by the first author on the same day to reflect intra‐operator variability. The first one was performed before their scheduled antenatal care, and the second one was performed after. Each examination was performed within 30 min as recommended by the British Medical Ultrasound Society 3rd Trimester Special Interest Group [20]. Real‐time images of the umbilical cord diameter were cross‐sectionally acquired and measured. In measuring the umbilical cord, it was tracked from the implantation point of the placenta, along its length, to the insertion of the fetus to assess for abnormalities. Real‐time images of the umbilical cord were cross‐sectionally acquired. The measurements of the three dimensions of the umbilical cord relevant to this study, namely, UCD, UCC, and UCA, were acquired using the same images. The callipers (measuring instrument on the ultrasound machine) were placed from one outer border to the other outer borders of the cord, in the cross‐sectional plane, to acquire the UCD (Figure 1).

**Figure 2:**
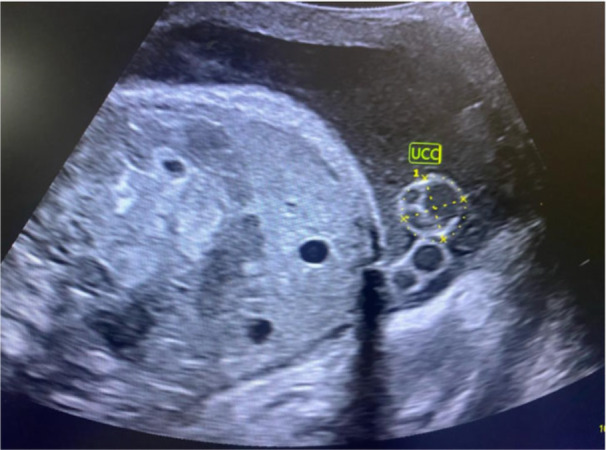
Sonographic measurement of umbilical cord circumference.

The value acquired for UCD was then divided by two to provide the radius. UCA was calculated from the formula: $A=\pi r^{2}$, where “A” represents the UCA, π is a constant, and *r* is the radius. Finally, the ellipse function of the calliper was used to obtain the UCC tracing the umbilical cord at its outer margins (Figure 2).

All UCMs were taken 2 cm away from the level of the cord insertion of the fetus. Birth weight (in kg) was measured immediately after delivery by physicians and midwives and then documented in the hospital's patient directory. The first author later retrieved and recorded this information.

### 2.8 Statistical Analysis

The data was cleaned, coded, and entered in the Statistical Package for the Social Sciences (SPSS) version 26.0 (IBM Corp., USA) for macOS. Data analysis included descriptive statistics of research variables and regression analysis. A preliminary evaluation of the data using the Kolmogorov–Smirnov test was conducted, and the results indicated a violation of the assumption of normality (the data were not normally distributed). Subsequently, the non‐parametric Spearman's correlation analysis was used to determine the correlation between birth weight and umbilical cord measurements. The strength of the association (the correlation coefficient, *r*) was interpreted based on Cohen's *r* interpretation: the correlation is small if *r* < 0.3, moderate if *r* = 0.5, and strong if *r* > 0.7 [21]. Cronbach's alpha coefficient (*α*) was used to assess intra‐operator variability between the two‐time UCD sonographic measurements. With a range of 0 to 1, a Cronbach's *α* value of 0.7 or more means that the measurements are highly consistent. A *p* value of less than 0.05 significant level was considered statistically significant at a 95% confidence interval.

### 2.9 Ethical Considerations

The study was approved by the Research Ethics Committee (REC) of the University of Health and Allied Sciences, Ho, Ghana (Reference number: UHAS‐REC A.1 (75) 23‐24). Subsequently, permission to undertake the study at the Ho Teaching Hospital (HTH) was granted by the Heads of the Radiology and Obstetrics and Gynaecology departments. All study participants gave informed written consent prior to study entry. To ensure privacy during recruitment, the aim and requirements of the study were explained in a private room within the antenatal clinic. To ensure privacy during the scanning process, access to the pregnant women was limited to only the first author. However, the pregnant women were allowed to have any other person they deemed fit to be in the room. The maternal characteristics, ultrasound measurements and birth weight of the neonates were anonymised. Also, all data collected for this study was limited to the research team only. This allowed the researchers to ensure confidentiality. The study protocol conformed to the Declaration of Helsinki.

## 3 Results

### 3.1 Descriptive Statistics

Maternal characteristics (age, gravida, and parity), obstetric parameters (estimated fetal weight and UCD), and birth weight were recorded. Out of the 107 pregnant women recruited, 97 completed the entire study, representing a response rate of 90.7%. The ages of the pregnant women ranged from 17 to 41 years, with a mean of 29.59 ± 4.83 years. Maternal data, obstetric measurements, and birth weight are illustrated in Table 1.

**Table 1:** Descriptive statistics of maternal characteristics, obstetric parameters, and birth weight.

Variable	Mean ± standard deviation	Range (min–max)
Maternal age (years)	29.59 ± 4.83	17–41
Gravida	2.76 ± 1.15	1–5
Parity	1.42 ± 1.01	0–4
Estimated fetal weight (kg)	3.41 ± 0.28	2.9–4.3
Birth weight (kg)	3.13 ± 0.27	2.6–3.8
Umbilical cord diameter (cm)	1.53 ± 0.19	1.17–2.22
Umbilical cord circumference (cm)	5.57 ± 0.41	4.85–6.81
Umbilical cord cross‐sectional area (cm^2^)	1.86 ± 0.47	1.07–3.89

### 3.2 Normality Testing

A Kolmogorov–Smirnov test was conducted on the different variables: maternal age, estimated fetal weight (EFW), birth weight (BW), umbilical cord diameter (UCD), umbilical cord circumference (UCC), and umbilical cord area (UCA). The results of the test are shown in Table 2. The results of the Kolmogorov–Smirnov test showed that the data for all of the variables except UCD and UCA are not normally distributed (*p* < 0.05).

**Table 2:** Normality test results.

Variable	Kolmogorov–Smirnov	
	Statistic	*p* value
Age (years)	0.107	0.009
Gravida	0.169	0.000
Parity	0.219	0.000
Estimated fetal weight (kg)	0.129	0.000
Birth weight (kg)	0.136	0.000
Umbilical cord diameter (cm)	0.061	0.200*
Umbilical cord circumference (cm)	0.106	0.009
Umbilical cord cross‐sectional area (cm^2^)	0.068	0.200*

*not significant.

### 3.3 Intra‐Observer Reliability Analysis

The Cronbach's alpha coefficient (*α*) was used to assess intra‐observer reliability. For each of the two UCD and UCC values, the measurements did not vary significantly, indicating strong consistency and precision in the measurements. Table 3 shows the intra‐observer measurement reliability assessment across all participants.

**Table 3:** Intra‐observer reliability analysis.

Variable	Mean ± SD		Cronbach's *α*	ICC (95% CI)
	First observation	Second observation		
Umbilical cord diameter (cm)	1.531 ± 0.185	1.524 ± 0.201	0.934	0.934 (0.901–0.956)
Umbilical cord circumference (cm)	5.573 ± 0.407	5.572 ± 0.417	0.976	0.976 (0.965–0.984)
Umbilical cord cross‐sectional area (cm^2^)	1.869 ± 0.459	1.844 ± 0.512	0.921	0.921 (0.882–0.947)

### 3.4 Correlation Between UCD and Birth Weight

**Figure 3:**
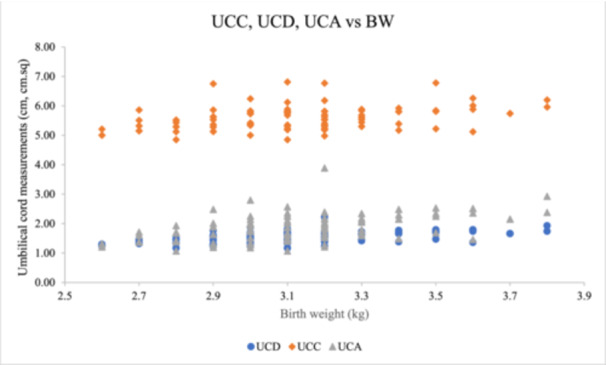
Correlation between umbilical cord measurements and birth weight.

A Spearman's correlation analysis between UCD and birth weight showed a moderate but statistically significant positive association (*r *= 0.53, *p* < 0.01, 95% CI = 0.346–0.674) (Figure 3). However, there was a low shared variance (*R* ^2^ = 28.0%) between the two variables from the regression analysis. A regression equation was formulated: UCD = *m *× BW + C, where *m* is the slope, and *C* is the intercept of the regression fit on the UCD axis.

### 3.5 Inferential Test for Statistical Significance Between UCC and Birth Weight

There was a medium (moderate), positive correlation between UCC and birth weight, where *r *= 0.331, *n *= 97, *p *< 0.001, 95% CI = 0.099–0.523, determined using Spearman's correlation. The result showed that UCC has a moderate relationship with BW. Also, it was noted that the coefficient of determination showed an 11.0% shared variance (Figure 3).

### 3.6 Inferential Test for Statistical Significance Between UCA and Birth Weight

There was a moderate (medium), positive correlation between UCA and birth weight, where *r* = 0.52, *n* = 97, *p*< 0.001, 95% CI = 0.363–0.656, determined from a Spearman's correlation. The result showed that UCA has a strong relationship with BW. Also, it was noted that the coefficient of determination showed a 27.5% shared variance (Figure 3).

### 3.7 Correlation Between Maternal Demographics and Umbilical Cord Measurements

The study investigated correlations between some maternal demographics and the various umbilical cord measurements using a Spearman rho correlation analysis. There were small positive correlations between UCA and all three demographics under study. Table 4 shows a breakdown of the correlation between maternal demographics and umbilical cord measurements.

**Table 4:** Correlation between maternal demographics and umbilical cord measurements.

Umbilical cord diameter			
Maternal demographics	*r* value	*p* value	Interpretation
Maternal age (years)	0.30	0.003	Moderate positive correlation
Gravida	0.26	0.009	Small positive correlation
Parity	0.26	0.009	Small positive correlation
**Umbilical cord circumference**			
Maternal age (years)	0.19	0.068	No correlation
Gravida	0.12	0.245	No correlation
Parity	0.13	0.217	No correlation
**Umbilical cord area**			
Maternal age (years)	0.29	0.004	Small positive correlation
Gravida	0.29	0.004	Small positive correlation
Parity	0.29	0.004	Small positive correlation

### 3.8 Multiple Regression Analysis

Multiple regression analysis of the predictors of birth weights in the study population was conducted. The model as a whole was significant in predicting neonatal birth weights among the study participants at F (4, 92) = 20.731, *p *< 0.001. The *R* ^2^ for the overall model for BW was 68.9% with an adjusted *R* ^2^ of 45.1%. A moderate effect size is reported by the model of variations in (BW) of the neonates in this study and is accounted for by the linear combination of the predictor variables (maternal age, parity, UCC, and UCD). These four variables correlated the most with BW and had high interdependent variable correlation.

Similarly, UCA and UCD showed a similar correlation to BW, with UCD having a higher correlation coefficient, thus leading to its inclusion. UCC was also included, as it was not dependent on UCD for its recorded values. In the final model, some of the independent variables were found to be statistically significant, and others were not significant with maternal characteristics.

Age (*t* = 3.501, *p* = 0.001, *β* = 0.371), parity (*t* = 1.104, *p* = 0.273, *β* = 0.116), UCC (*t* = −0.858, *p* = 0.393, *β* = −0.101) and UCD (*t* = 3.786, *p* < 0.001, *β *= 0.459) (Table 5) in predicting BW. The final predictive equation of the model for predicting BW was given as:

BW = 1.849 + maternal age (0.021) + parity (0.031) + UCC (−0.066) + UCD (0.652).

**Table 5:** Multiple regression analysis.

Independent variables		95% CI					Correlations	
	*B*	Lower	Upper	*β*	*t*	*p*	Partial	Part (Sr2)
(Constant)	1.849	1.219	2.479		5.830	0.000		
Maternal age (age)	0.021	0.009	0.032	0.371	3.501	0.001	0.343	0.265
Parity	0.031	−0.025	0.086	0.116	1.104	0.273	0.114	0.083
UCC (cm)	−0.066	−0.220	0.087	−0.101	−0.858	0.393	−0.089	−0.065
UCD (cm)	0.652	0.310	0.994	0.459	3.786	0.000	0.367	0.286

*Note:* Model summary.

*R* ^2^ = 0.689, adj. *R* ^2^ = 0.451, *p* < 0.01, ANOVA: df (4,92) = 20.731, *p* < 0.01.

## 4 Discussion

The mean age of the pregnant women was 29.59 ± 4.83 years (Table 1). This was quite similar to the mean ages recorded by studies conducted in India (25 ± 4 years) [21], Iran (27 ± 5.5 years) [22], and Nigeria 29 ± 6 years [23]. It has been observed that the mean maternal age among women of childbearing age has been increasing globally [24]. This trend recorded in our study could be attributed to most of the pregnant women being residents of the regional capital (location of the hospital) and being either educated or engaged in various economic activities, thereby influencing their decisions to have children. This assertion has also been observed in the United States [24] and Australia [25]. Major contributory factors to this increasing trend in maternal age among first‐time mothers include societal, cultural and life modifications, increased use of assisted reproductive technologies, and planned age of childbirth for reasons such as women's empowerment, educational attainment, and professional development [26, 27]. Maternal age tends to influence pregnancy outcomes. The study showed a strong positive correlation between maternal age and birth weight. This result means that birth weight increases with maternal age. However, it was not within the scope of this study to assess the influence of maternal age on UCD. This finding suggests that further studies may be warranted to establish any association or lack thereof.

### 4.1 Umbilical Cord Measurements

The mean UCMs measured from the pregnant women was 1.53 ± 0.19 cm (Table 1). The mean UCD compares with those reported in Nigeria by Udoh et al. [28] and in Sudan by Elghazaly et al. [29] as 1.59 ± 0.18 cm and 1.50 cm, respectively. The direct similarities among these studies could be ascribed to the studies being conducted among black populations. Birth weight plays a key role in neonatal health by providing vital information about the health status of the newborn. The mean birth weight of newborns in this study was 3.13 ± 0.27 kg (ranging from 2.6 to 3.8 kg). Similar birth weights have been reported in Cape Coast (3.25 ± 0.52 kg) [30] and Ho (3.03 ± 0.57 kg) [31], both within Ghana. Birth weights within this range are generally thought to be normal and are indicative of quality antenatal care and healthy motherhood among the sampled women in the respective studies. There was a moderate but statistically significant positive correlation between birth weight and UCD. A positive relationship between UCD and birth weight has previously been reported [32, 33], highlighting the relevance of UCD in the prediction of birth weight using the regression equation derived from the best‐fitting line to the scatterplot of the data. The same kind of relationship was noted for UCA and BW. Between UCC and BW, however, a medium positive correlation was noted. The similarity in values of UCD and UCA could be due to the UCA being dependent on the UCD. UCD correlates with birth weight because it reflects the structural and functional capacity of the fetoplacental circulation, which directly influences fetal growth [34]. Similarly, UCA correlates with birth weight because it integrates multiple anatomical and physiological determinants of fetoplacental function that are central to fetal growth. Compared with diameter alone, cord area more comprehensively reflects the cord's capacity to support blood flow and nutrient exchange [1]. UCC may fail to correlate with birth weight because it is a relatively crude geometric and biological measure that does not reliably capture the functional determinants of fetal growth. Circumference reflects only the outer boundary of the cord and does not directly quantify vascular lumen size, total cross‐sectional area, and Wharton's jelly volume. This showed that UCD and UCA correlate more strongly with birth weight compared to UCC. The clinical interpretation of the association is that a larger umbilical cord diameter and circumference could be associated with a heavier baby.

### 4.2 Predicting Birth Weight With UCM and Maternal Characteristics

In this study, multiple regression analysis, factoring in maternal characteristics, produced the formula BW=1.849+maternalage(0.021)+parity(0.031)+UCC(−0.066)+UCD (Table 5). A study by Nahum and Stanislaw [35] evaluated 25 ultrasonic algorithms and a maternal characteristics‐based equation for predicting term birth weight, finding that ultrasonic methods had correlations with actual birth weight ranging from 0.44 to 0.79, while the maternal characteristics equation had a correlation of 0.60 and similar prediction errors (~± 353 g) to the best ultrasound algorithms. Their findings suggest that ultrasonographic fetal biometric measurements do not significantly outperform maternal characteristic‐based predictions. This current study's multiple regression formula incorporates maternal age, parity, UCC, and UCD and offers a novel approach by including umbilical cord measurements, which are not usually put together in other predictions. Furthermore, the two most dependable UCMs are UCC and UCD, which had a statistically significant correlation with birth weight for both. As a result, the suggested formula is more robust since it takes into account maternal characteristics when predicting birth weight. The clinical implication is a shorter procedure for estimating fetal weight as this formula requires recording measurements from one image. This will reduce the downtime required to estimate weight compared to the orthodox EFW measurements, which would be especially useful in an emergency setting. Weights of foetuses with abnormal biometric characteristics can also be assessed. The most effective formulae for use in the clinical context will be determined by comparing the various formulae based on their predictive strength.

## 5 Limitations

The accuracy of the measured umbilical cord diameter from the ultrasound machine depends on the skill level of the operator and the calibration of the machine. Measurement errors could have been introduced by variations in the measurement procedure used by the operator(s) and/or the equipment calibration. The potential effect of these errors on the results was not addressed. In addition, the population was small, which affected the generalizability of the data. Future studies could increase the size and diversity of the study populations by extending the research to include a wider range of mother age groups, ethnic groupings, and socioeconomic backgrounds. This will facilitate the creation of population‐specific reference ranges and offer a more thorough knowledge of the link between UCMs and BW in various circumstances. Future studies should also consider a longitudinal study to monitor changes in umbilical cord growth during pregnancy by tracking gestational age from 14 weeks to term. This will improve the accuracy of birth weight prediction and assist in uncovering potential changes in umbilical cord measurements at different gestational ages.

## 6 Conclusion

This baseline cross‐sectional study examined the relationship between umbilical cord measurements (UCMs) and birth weight among term pregnant women in a teaching hospital in Ghana. The significant correlations observed between birth weight and UCMs suggest that umbilical cord measurements may provide useful information for assessing fetal growth, including in structurally atypical foetuses such as those with hydrocephalus, achondroplasia, dwarfism, gastroschisis, or omphalocele. However, while the correlations were statistically significant, their predictive strength is modest, and the findings primarily demonstrate associations rather than strong predictive capacity. The modest, less diverse sample size and single‐centre design of the study, however, limit the generalizability of these findings.

## Author Contributions

**Swallah Alhaji Suraka:** conceptualization, investigation, writing – original draft, writing – review and editing, methodology, resources, data curation, validation. **Abdul Nashirudeen Mumuni:** writing – original draft, writing – review and editing, and supervision. **Nathaniel Awentiirin Angaag:** writing – original draft, writing – review and editing, and visualization. **Larisa ‐ Ioana Coman:** writing – original draft, writing – review and editing. **Eric Kwasi Ofori:** writing – review and editing and supervision.

## Funding

The authors have nothing to report.

## Conflicts of Interest

The authors declare no conflicts of interest.

## Transparency Statement

The lead author, Nathaniel Awentiirin Angaag, affirms that this manuscript is an honest, accurate, and transparent account of the study being reported; that no important aspects of the study have been omitted; and that any discrepancies from the study as planned (and, if relevant, registered) have been explained.

## Data Availability

The data that support the findings of this study are available on request from the corresponding author. The data are not publicly available due to privacy or ethical restrictions.
